# Fungicide Resistance Evolution and Detection in Plant Pathogens: *Plasmopara viticola* as a Case Study

**DOI:** 10.3390/microorganisms9010119

**Published:** 2021-01-06

**Authors:** Federico Massi, Stefano F. F. Torriani, Lorenzo Borghi, Silvia L. Toffolatti

**Affiliations:** 1 Dipartimento di Scienze Agrarie e Ambientali, Università degli Studi di Milano, Via Celoria 2, 20133 Milano, Italy; 2 Syngenta Crop Protection Münchwilen AG, 4334 Stein, Switzerland; stefano.torriani@syngenta.com (S.F.F.T.); lorenzo.borghi@syngenta.com (L.B.)

**Keywords:** grapevine, downy mildew, oomycete, fungicide resistance

## Abstract

The use of single-site fungicides to control plant pathogens in the agroecosystem can be associated with an increased selection of resistance. The evolution of resistance represents one of the biggest challenges in disease control. In vineyards, frequent applications of fungicides are carried out every season for multiple years. The agronomic risk of developing fungicide resistance is, therefore, high. *Plasmopara viticola*, the causal agent of grapevine downy mildew, is a high risk pathogen associated with the development of fungicide resistance. *P. viticola* has developed resistance to most of the fungicide classes used and constitutes one of the most important threats for grapevine production. The goals of this review are to describe fungicide resistance evolution in *P. viticola* populations and how to conduct proper monitoring activities. Different methods have been developed for phenotyping and genotyping *P. viticola* for fungicide resistance and the different phases of resistance evolution and life cycles of the pathogen are discussed, to provide a full monitoring toolkit to limit the spread of resistance. A detailed revision of the available tools will help in shaping and harmonizing the monitoring activities between countries and organizations.

## 1. *Plasmopara viticola*: Characteristics and Management

**Figure 1:**
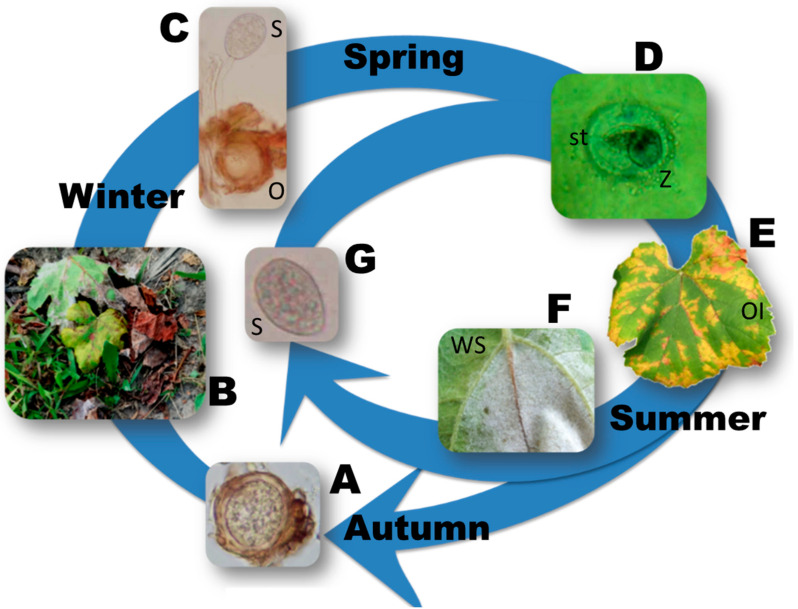
Disease cycle of *P. viticola*: the pathogen survives the winter period as oospores, i.e., the overwintering structures differentiated by sexual reproduction in autumn (**A**), embedded in dead leaves on the vineyard floor (**B**). With favorable weather conditions, oospores typically produce sporangia (**C**) that, in turn, produce zoospores (**D**). Zoospores are splashed by rain onto leaves and other receptive tissues of the grapevines, originating the primary infections through stomata penetration (**D**). Disease symptoms, visible as yellow discoloration (oil spots, Ol) on the upper side of the leaves (**E**), appear at the end of the incubation period and are followed, in high humidity conditions, by the emission of sporangiophores (**F**) with sporangia (**G**) that will cause secondary infections through the emission of new zoospores. O = oospore; S = sporangium; st = stoma; Z = zoospore; OI = oil spot symptom on the upper side of the leaf; WS = white sporulation, consisting of sporangiophores and sporangia, on the underside of the leaf.

Downy mildew, caused by the oomycete *Plasmopara viticola*, is one of the major threats for grapevine production, due to the quantitative and qualitative yield losses that are associated with severe disease epidemics [1]. *P. viticola* is an obligate parasite of grapevine, causing the main damage to the Eurasian grapevine species (*Vitis vinifera*), which is the most cultivated species worldwide due to the high quality of its grapes. Most of the *V. vinifera* cultivars are highly susceptible to the pathogen, and only recently have sources of resistance been found in the center of origin of viticulture, which is located in Georgia (South Caucasus) [2,3]. This high susceptibility makes chemical control of the pathogen the most important measure to ensure an adequate yield. The timing of fungicide application depends on pathogen features and on weather conditions. *P. viticola* is a polycyclic pathogen, able to undergo numerous infection cycles during a single grapevine growing season. It overwinters as oospores (Figure 1A), which are sexual structures found in dead leaves on the vineyard floor (Figure 1B). In spring, with favorable weather conditions, oospores produce a single macrosporangium (Figure 1C), where the asexual spores (the zoospores) are formed. The zoospores infect the receptive grapevine tissues through stomata (Figure 1D) in the presence of free water, provided by rain or dew, at temperatures below 32 °C. Consequently, frequent fungicide applications are needed in vineyards located in areas with frequent rainfall and moderate temperatures during the grapevine growing season [4].

## 2. The History of the Chemical Control of *P. viticola*

From the end of the Nineteenth Century, when the first agrochemical compounds were tested against *P. viticola*, until now, the panorama of phytoiatric practices has changed greatly, especially because of the availability of new active substances. Although agronomic practices represent a useful tool for disease management and the development of resistant varieties has made great progress, the use of chemical products still represents today the only effective means to control this fungal disease [5]. The growing of traditional varieties of *Vitis vinifera* is not conceivable without the use of fungicide applications [6]. The first documented attempts to control downy mildew using chemicals dates back to 1882, when the French botanist Pierre-Marie-Alexis Millardet noticed that the grapevine plants cultivated along the roadside did not show *P. viticola* symptoms. In the field, only these plants were treated, with a mush made with copper sulphate and lime, to discourage people from eating the grapes. This observation led to the development of the “Bordeaux mixture” to control downy mildew [7]. Its strong efficacy in inhibiting multiple metabolic processes in the fungal pathogen, together with a robust fastness and persistence, made the Bordeaux mixture quickly popular first in Europe, then in Australia and the USA [8]. Among protectant fungicides, copper still represents the most traditional and used chemical. However, intensive use of copper can cause serious environmental problems such as accumulation in the soil and adverse negative effects on beneficial organisms.

The use of the Bordeaux mixture in agriculture was greatly reduced during the Second World War, because copper was preferentially needed by the weapon industries [9], and its availability for agriculture became secondary. Alternative control compounds were evaluated, but the results were always disappointing [10]. Experiments were conducted using zinc, aluminum, magnesium sulphates, and other metal salts, such as iron, silver, cadmium, and chromium. After several years of testing, the conclusion was that there were no better alternatives to the Bordeaux mixture [11]. Because of the scarcity of copper and the absence of options, growers started preparing the Bordeaux mixture with a lower concentration of copper sulphate. Despite the lower dose, disease control was still acceptable in many cases, if the fungicide was employed at the right time during the epidemics. This highlighted the importance of correct and timely applications [12].

After the Second World War, the first organic fungicides were synthesized by the chemical industry to control downy mildew. The dithiocarbamates and phthalimides were the first chemical classes employed against *P. viticola*. Members of these classes (e.g., zineb and captan), showed similar or higher control than the Bordeaux mixture [13,14]. The success of these fungicides was mainly caused by the higher return on investment and the absence of phytotoxicity, the latter often observed when using copper compounds [15,16]. However, intensive use of dithiocarbamates induced an excessive vegetative growth, favoring infections by other pathogens such as *Botrytis cinerea*, the grey mold agent [5,6,17,18]. Environmental toxicity and interference with natural competitors of spider mites like *Tetranychus urticae* and *Panonychus ulmi* [19,20] were reported as well.

A second wave in the development of control solutions occurred between the 1970s and the 1980s, when target-site fungicides were introduced into the market. Target-site fungicides inhibit a single biochemical pathway within the fungal cell [21] and generally have a more favorable toxicological profile compared to previous, multisite solutions, which interfere with numerous metabolic processes of the fungus [22,23,24]. Many of the newly discovered fungicide classes were systemic or cytotropic, i.e., able to penetrate and redistribute in the plant tissues, ensuring a better rain fastness and curative activity [25]. The substantial difference between systemic and cytotropic active ingredients is that the former can translocate inside the tissues of the plant (mainly through xylem vessels) and protect the newly formed vegetation, whereas the latter redistribute only locally [23].

## 3. Fungicide Resistance: A Threat to Downy Mildew Control

With the introduction of target-site fungicides, a new threat soon appeared in downy mildew control: fungicide resistance. Fungicide resistance can be defined as the acquired and heritable reduction in the sensitivity of a fungus to a specific anti-fungal agent (Background Information, www.frac.info). Normally, plant pathogen populations are characterized by a low frequency of resistant individuals that do not interfere with disease control in the open field. Problems with disease control can occur when resistant individuals become predominant over sensitive individuals. The evolution of fungicide resistance in a population is determined by the interaction of different factors, such as the fungicide’s mode of action and utilization, the pathogen biology and epidemiology, and the agronomic practices adopted in the field. In the following paragraphs, these factors will be described more in detail and indications on the management of resistance through ad-hoc strategies, aiming at reducing resistance evolution, will be provided, using *P. viticola* and grapevine as a model system.

Fungi and fungal-like organisms such as the oomycetes, where *P. viticola* belongs, share a great capacity of evolution because of their high reproductive frequency [26]. *P. viticola* is a high risk pathogen because of its complex life cycle, which includes sexual and asexual reproduction and polycyclic behaviors (Figure 1) [27]. The genetic changes that might occur after each reproductive cycle are probably disadvantageous or neutral. However, in some cases, they can provide a fitness advantage [24]. Fungicide resistance occurs when one of these genetic mutations leads to a stable and heritable reduction in sensitivity to a specific fungicide [28]. Following repeated treatments with the identical active substance, which exerts a selection pressure on the fungal population [29], the percentage of sensitive individuals can decrease in favor of resistant mutants. When resistant mutants turn dominant in the population, the pathogen can no longer be adequately controlled by the fungicide [30]. Fungicides that share the same mode of action should be considered cross-resistant since they inhibit the same target and should not be used without recommendations, thus avoiding the selection of resistant populations [31].

Generally, fungicide resistance can be conferred by five major mechanism: (i) alterations in the target site that decrease binding to the fungicide; (ii) overproduction of the target protein; (iii) presence of an alternative metabolic pathway capable of bypassing the process inhibited by the fungicide; (iv) metabolic breakdown of the fungicide; and (v) active export or exclusion of the fungicide [31,32,33]. The resistance mechanisms known for *P. viticola* can be found in the references listed in Table 1.

Resistance emerged soon after the introduction of systemic and cytotropic products, from the 1970s onwards [24,34]. The substantial difference between systemic and cytotropic active ingredients is that the former can translocate inside the tissues of the plant (mainly through xylem vessels) and protect the newly formed vegetation, whereas the latter only redistribute locally [23]. This happened because, compared to multisite fungicides that interfere with many different metabolic processes, the new molecules were prevalently single-site or site-specific fungicides [31]. In the case of targeted fungicides, single nucleotide polymorphisms (SNPs) in the gene encoding for the fungicide target could cause decreased sensitivity. Multisite fungicides, on the other hand, are associated with a lower risk of resistance evolution since several mutations would need to occur simultaneously in different genes in order to prevent the fungicide from binding to its multiple targets [31].

**Figure 2:**
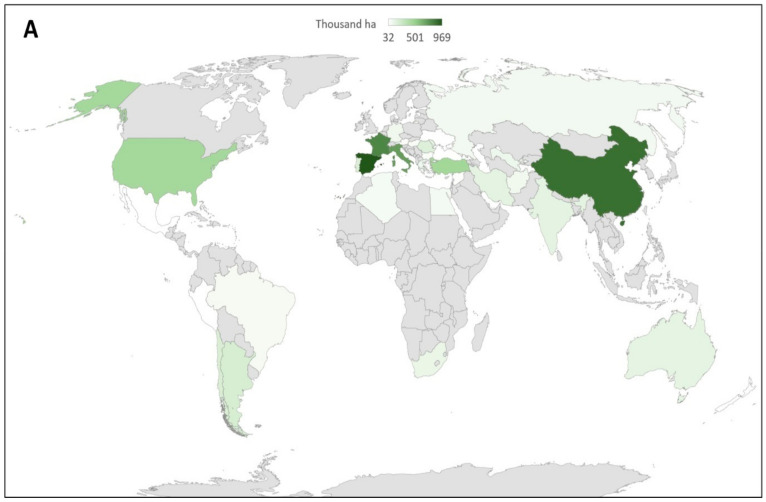

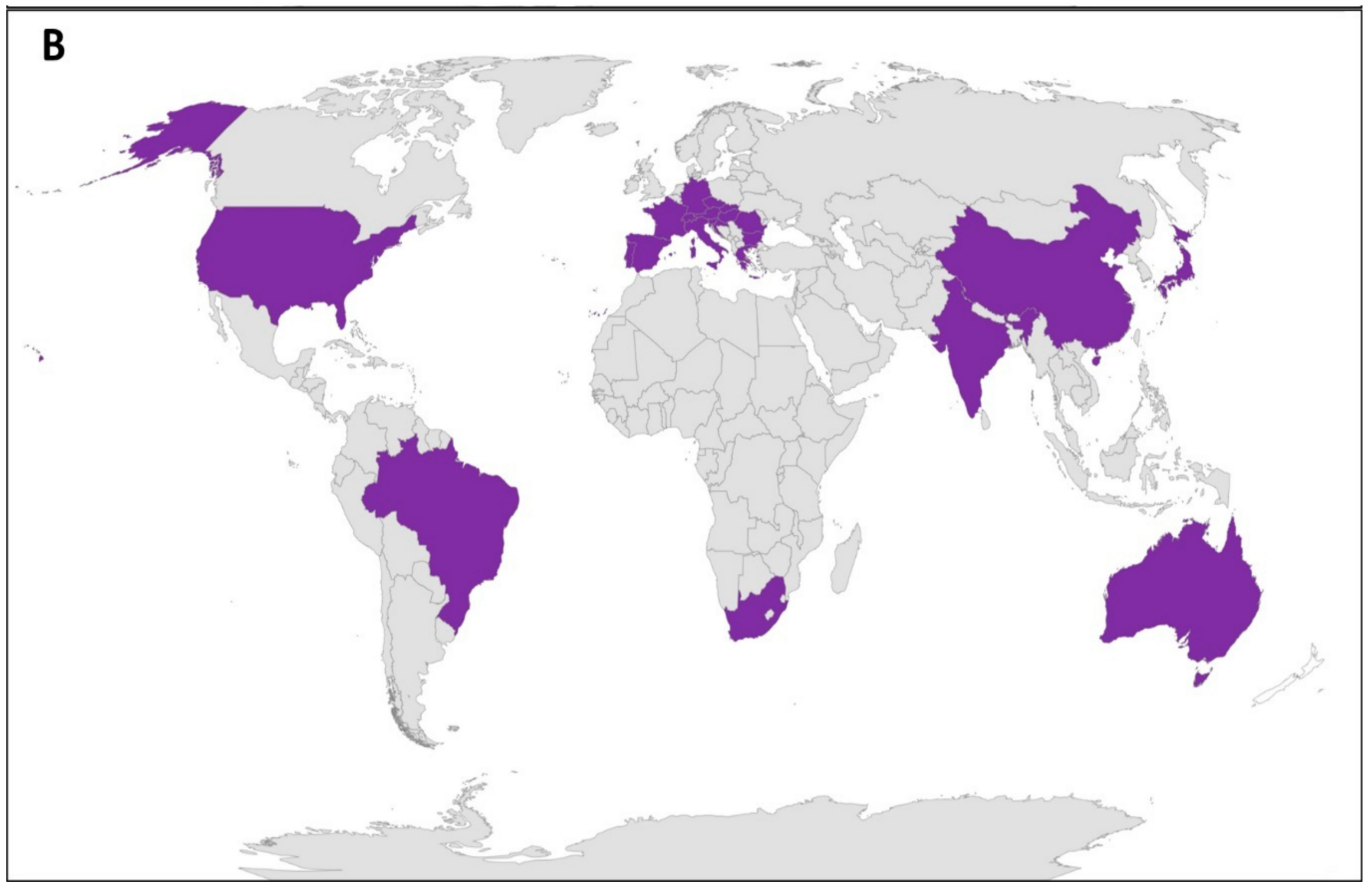
Global vine-growing areas allocated for the production of wine grapes, table grapes, or dried grapes in 2018 (sources Organisation of Vine and Wine and ood and Agriculture Organization of the United Nations) (**A**), compared to countries where *P. viticola* fungicide resistance was reported in 2020 (**B**) [34,35,36,37,38,39,40,41,42,43,44].

Resistance to different fungicide modes of action in *P. viticola* has been reported (Table 1) in the main vine-growing areas (Figure 2) using different detection techniques [34,35,36,37,38,39,40,41,42,43,44].

## 4. Fungicide Resistance Management

The definition of a balanced fungicide strategy accounting for good disease control and preventing resistance progress is the current challenge. The repeated use of solo fungicides with a single-site mode of action is often associated with a higher risk of resistance evolution when compared to a more diversified approach, e.g., multiple fungicide classes in mixtures or in alternation [45]. Anti-resistance strategies are valued in sustainable agriculture since they aim to control the disease and reduce the selection of fungicide resistance. The target of fungicide resistance management is to decrease the selection and diffusion of resistant genotypes in natural populations, as described by the reduction of the selection coefficient [45,46]. This value is determined by the combination of the selective advantage of the resistant strains in the presence of the fungicide and the potential fitness cost associated with resistance in the absence of selection (i.e., negative selection, associated with decreased fitness). Fitness is measured by the per capita rate of increase of the resistant and sensitive strains of a population [24,45]. The goal of practical management is the reduction of the selection coefficient (i.e., the selection pressure), thus maintaining an acceptable level of disease control and avoiding yield losses [47].

Grapevine is a perennial plant with a life expectancy of decades; it is cultivated in monoculture, with a period of susceptibility to *P. viticola* of several months that varies each year. It is clear how delicate the management of fungicide resistance for this crop is. The agronomic risk of selecting for resistance associated with vineyards is high, because numerous fungicide sprays are needed every season [48].

Anti-resistance recommendations can be summarized as follows: use of fungicide mixtures belonging to different classes; avoidance of curative and eradicative applications since they do not allow an adequate control of the pathogen diffusion, which is guaranteed only by preventive fungicide treatments; limitation of the number of treatments per season; application of the fungicide only when strictly required following the recommended dose [49]. In the specific case of grapevine downy mildew, because of the high pathogenic and agronomic risks, the implementation of correct anti-resistance strategies is challenging [50] and must consider local variations in fungicide sensitivity. The generation of local recommendations, based on specific population sensitivity profiles, requires conducting the organization and carrying out of effective and validated monitoring programs and allow the best application timing in relation to pathogen development [47].

Resistance spread has practical consequences when the lower sensitivity of the pathogen to a fungicide leads to the reduction or loss of disease control in the field (practical resistance). In the worst case, resistance emergence can lead to usage restriction or even suspension of those fungicides with high resistance risk [50,51,52]. Fungicide resistance reports annually published by FRAC (Fungicide Resistance Action Committee) must be, therefore, carefully interpreted and the recommendations followed in order to avoid practical resistance issues. Still, small changes in sensitivity to fungicides or the rate of resistant individuals at a low frequency have sometimes been overestimated [31]. The confirmed presence of a strain showing decreased sensitivity to a fungicide is not necessarily linked to a reduced control of the disease in vineyards. Studies conducted in the laboratory on *P. viticola* sporangia isolates and artificial mixed sporangia populations demonstrated that, in some cases, similar conclusions on fungicide resistance could be drawn with sporangia suspensions containing 1% or 100% resistant sporangia [53]. Furthermore, identical *P. viticola* populations tested with different methodologies can generate different results. On the other hand, failures in detecting resistance can be attributed to the choice of methods that are inefficient at quantifying low rates of resistant phenotypes [54]. To limit such false positive and negative issues, the development of standardized, quantitative, reproducible, and readily understandable testing methods has been a primary goal of several organizations such as EPPO (European and Mediterranean Plant Protection Organization) and FRAC. Still, the proper evaluation of the pros and cons of different proposed methods needs years of validation, and not all procedures have the same power when scoring fungicide resistance to different modes of action [55].

## 5. Strategies for Monitoring Fungicide Resistance

The degree of success of anti-resistance strategies is strongly influenced by the timing of the start of the monitoring activity [56]. Resistance monitoring allows detecting changes in the sensitivity of a pest population subjected to different disease pressure levels and spray programs, over several years and in different locations [57]. This activity is usually performed at the national or regional levels, but also by technical world-wide associations such as FRAC [31]. What often happens is that monitoring tends to start after indications of decreased sensitivity in the field. As a consequence, monitoring data are not obtained early enough to allow any possible action to preserve the efficacy of the affected product. The initial assessment of the natural range of sensitivity of the pathogen towards the fungicide is, on the contrary, necessary for the interpretation of any shift in further monitoring activities [58]. In the past, this kind of information was rarely available, but recently, the agrochemical industry has become committed to presenting baseline sensitivity as part of the registration requirements [52].

**Figure 3:**
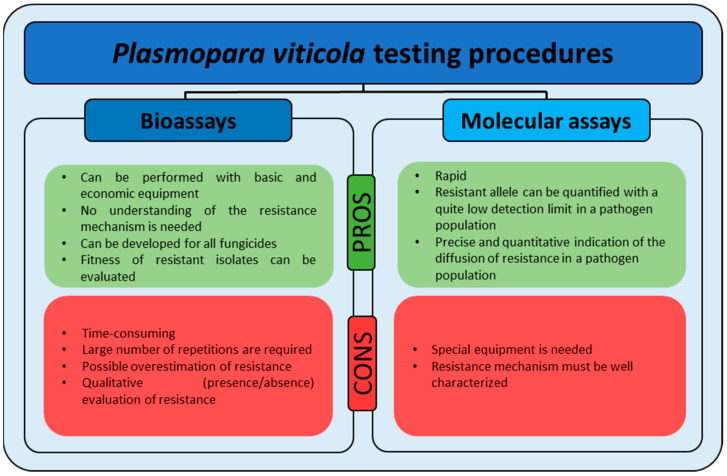
Advantages and disadvantages of biological and molecular assays that should be considered when choosing the testing method.

As with other organisms, the detection of resistance in a fungal population can be determined from the comparison between base-line data presented in the literature, which define the normal level of sensitivity of a population never exposed to a specific fungicide, and the data obtained from suspected resistant isolates [59,60,61]. The establishment of validated methods is the first crucial step to create a sensitivity baseline to enable comparisons with subsequent sensitivity data. Fungicide resistance is assessed with different methodologies that can be divided into two main categories: bioassays and molecular assays (Figure 3). Bioassays evaluate the response of the pathogen, in terms of growth and sporulation, to the action of the fungicide [62]. They can be developed for every fungal species with different levels of complexity (from simple growth on a synthetic medium, for cultivable species, to pathogenicity assessment, for uncultivable species) [59,63] and performed in laboratories with basic equipment. Bioassays have the advantage that the sensitivity profile is determined independently of the underlying mechanism of resistance. Their main disadvantages are the long time required to obtain results and the type of information provided: these methods often give a qualitative indication (presence/absence) of resistance occurrence, whereas proper anti-resistance strategies require quantitative information (e.g., percentage of resistant over sensitive individuals) on the pathogen population composition. Molecular assays are performed once the SNP(s) in the fungicide target gene, associated with resistance, is known and allow identification and quantification of the mutated alleles in a population, providing a quantitative indication of resistance rates [64]. An overview of the criteria and methods, from sampling to data interpretation, developed for monitoring fungicide resistance in *P. viticola* populations is reported in the next paragraphs.

## 6. Sampling

The first step of monitoring is field sampling. Two different sampling methods can be applied based on plant development or geography [56]. The two approaches are complementary: the first one gives an overall view of resistance at specific plant developmental stages, while the second one evaluates the spread of resistance in vineyards given different disease and treatments’ pressures [65]. Usually, *P. viticola* samplings are performed at a single stage, after the final fungicide spraying, between August and September. An alternative strategy consisting of multiple collection times, from the beginning to the end of the season, can be very useful for investigating the fitness of *P. viticola* resistant strains and the effects of specific treatments on the selection of the resistant sub-population [54,66,67].

At least 50 grapevine leaves with downy mildew symptoms are randomly collected from the vineyard or from specific vineyard plots. Immediately after harvesting, and until arrival in the laboratory, the leaves are preserved in cold conditions to avoid the degradation of the inoculum [4,54,66,68]. A critical success factor is related to the proper storage of the samples between collection and testing. It is very difficult to successfully store *P. viticola* on dried plant material; therefore, freezing the material for conservation could be considered. In this case, however, additional investigations with proper controls are needed to test whether or not the viability of the sample has been negatively affected [55].

## 7. Bioassays

A range of bioassay methods for monitoring fungicide resistance in *P. viticola* have been developed [62,69,70]. Since *P. viticola* is an obligate pathogen, it cannot be cultivated or propagated on synthetic media. As a consequence, the use of one of the most common bioassays employed for measuring fungicide sensitivity, the in vitro mycelium growth test on agarized media amended with fungicide, is not possible [59,63,71]. The most reliable approach to test obligate biotrophs is by experimentally inoculating the pathogen inoculum on entire plants (in planta assays) or detached leaves (in vitro assay) preventively treated with the fungicide of interest [72]. Sensitivity is usually measured by determining a toxicological parameter, the EC_50_, which represents the concentration of fungicide able to inhibit pathogen infection (estimated from the symptomatic area or the area covered by sporulation) by 50% compared to a negative control. By comparing the EC_50_ values of the monitored samples to those present in the baseline, it is possible to quantify shifting in sensitivity [31]. Monitoring the fungicide sensitivity of *P. viticola* through bioassays is time-consuming, as it requires sampling, isolation (facultative), and inoculation of the pathogen on living plant material [58,73]. This protocol involves a large number of repetitions to reduce the variability linked to the fact that different leaves can have a different interaction with the pathogen and requires a large production of plant material. Since the isolation of *P. viticola* is difficult and time-consuming, often bulks of strains are tested. This can lead to qualitative results, which tend to overestimate the resistance phenomenon because of the necessary use of high concentrations of spores in the process of artificial inoculation compared to field conditions [50].

The use of standardized methods and shared reference strains is essential to enable comparisons between different monitoring programs and labs. To achieve this purpose, FRAC published a catalogue of approved standardized methods sorted by pathogen and assay type that allow a direct comparison between results obtained at different research centers [70]. Here, we review a range of methods available to monitor fungicide resistance in *P. viticola* populations in relation to the different resistance evolutionary phases and life cycle of the pathogen. The choice of the test protocol should consider which fungicide, resistance evolutionary phases, and life cycle steps of the pathogen are under investigation. The different methodologies available in the literature to monitor *P. viticola* resistance are described below. Despite the great number of published methods, many of them have been grouped together because of their similarity.

### 7.1. In Vivo Assays

**Figure 4:**
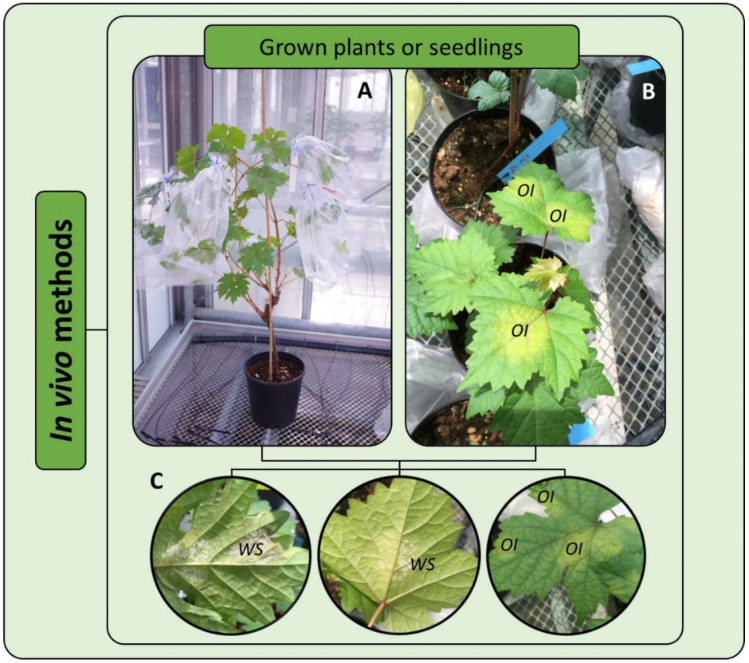
In vivo tests carried out on grapevine plants (**A**,**B**) aiming at assessing fungicide resistance through the evaluable 50 value of the *P. viticola* population. OI = oil spot symptom on the upper side of the leaf; WS = white sporulation, consisting of sporangiophores and sporangia, on the underside of the leaves.

In the case of *P. viticola*, as for other obligate biotrophs, in vivo tests that are carried out on adult plants or seedlings are challenging. A first issue is related to plant material production during the whole monitoring period that might require a significant logistic effort. The cost and time associated with plant production might be the limiting factors for a high-throughput experiment and can impact the possibility of including replicates, as is normally done for in vitro testing. Whole plant assays are based on the evaluation of pathogenicity on intact plants. The tested fungicide is applied at increasing rates to the leaves (usually the third–fifth from the apex of the shoot) using a laboratory sprayer. The fungicide must be uniformly applied to both the upper and lower side of the leaf one day prior to the inoculation of the sporangia suspension (5 × 10^4^ sporangia/mL) with a handheld sprayer. Formulated products should be preferred instead of the use of technical active ingredients, which might have issues relating to adherence to the plant surface. Inoculated plants are subsequently incubated in a climate chamber at 20 °C and saturating humidity (Figure 4A) for a period of six days, after which disease assessment is visually performed on three leaves per plant (four plants for treatment as biological or technical replicates) to compare the disease severity of the treated and untreated control samples (Figure 4B,C) [74]. In some cases, the same population tested using whole plant or detached leaf disc assays can generate different results [53]. It appears that changes in physiological and molecular states caused by leaf detachment can contribute to decreasing the host resistance response compared to that of intact plants [75,76]. Furthermore, it may be possible that whole plant assays are ineffective to detect a low proportion of resistant phenotypes [54].

### 7.2. In Vitro Assays

In vitro testing for obligate pathogens such as *P. viticola* are based on leaf disc inoculation or on spore germination assessment. The use of those techniques requires a great deal of organization, and for obligate pathogens, this test is usually performed in microtiter plates [55].

#### 7.2.1. Leaf Disc Assay

Many established in vitro tests based on plant tissues are available for *P. viticola* [77,78,79,80,81,82,83]. These methodologies are slightly different, such as for the size of the leaf discs and the way fungicide and inoculum are applied, but all of them allow the testing of large numbers of samples in a short time, using a miniaturized test where portions of the leaves are inoculated with the pathogen. The use of such methods has the great advantage of minimizing the costs in terms of time and resources compared to whole plant assays, and the tests are compatible with all fungicide classes, but in absolute terms, these tests remain highly resource-demanding [31]. Moreover, this type of method does not allow a precise evaluation of the percentage of resistant strains in the population tested, since the information they can provide is limited to a qualitative description of the resistance status.

**Figure 5:**
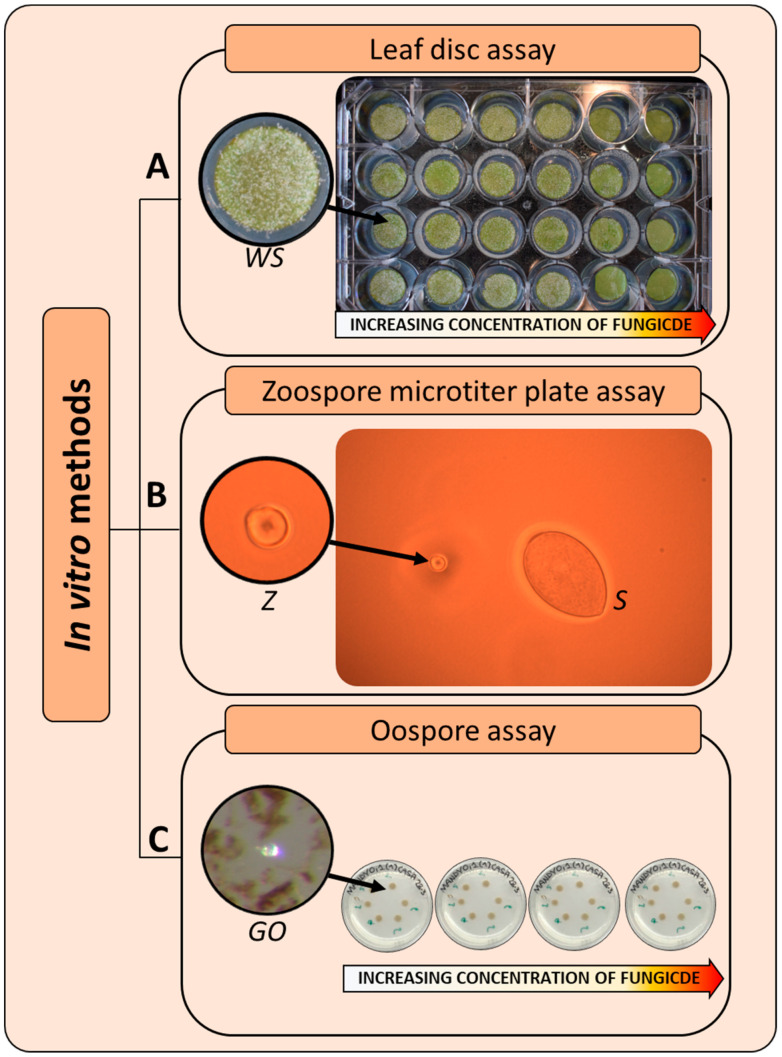
In vitro testing for *P. viticola* based on leaf disc bioassay (**A**), zoospore microtiter plates (**B**), and oospore testing (**C**). (**A**) microtiter plate containing leaf discs showing white sporulation (WS). Columns were treated with increasing concentrations of fungicide. (**B**) Sporangium (S) and free zoospore (Z) in liquid medium. (**C**) Agar plates containing increasing concentrations of fungicides and inoculated with oospore suspensions. The number of germinated oospores (GO) is counted and used to calculate the germination percentages at each concentration and to estimate the EC_50_ values of the population or the percentage of resistant oospores at a discriminatory concentration of fungicide.

Within this group, two of the approved standard methodologies by FRAC are included: the PLASVI microtiter plate test and PLASVI monitoring [82,83]. Considering that *P. viticola* is an obligate biotroph and that assays are often not performed directly on the collected samples, the former method implies propagation of the pathogen on fresh plant material. Collected sporangia are inoculated on new healthy grape leaves placed into a Petri dish containing filter paper soaked with water to prevent dehydration. The Petri dishes are then incubated at 19 °C with a 12 h:12 h photoperiod inside a plastic box containing soaked filter paper. Fresh sporangia are collected after seven days and resuspended in water, obtaining a sporangia suspension that will be sprayed onto the lower side of fresh healthy leaves using an atomizer. Sporangia suspensions should be standardized at a concentration of 5 × 10^4^ sporangia/mL and applied to 24 leaf discs of 15 mm in diameter placed in a 24-well plate and sprayed with fungicide 24 h before the inoculation (Figure 5A). The discs are incubated in a climate chamber for a period of six days, after which the assessment is visually done by determining the percentage of infected leaf area [82]. Normally, a range of fungicide concentrations is used in the test to generate an EC_50_ value. An alternative strategy consists of choosing a few discriminatory doses (i.e., doses of fungicides able to discriminate resistance from sensitivity) previously identified as relevant to describe a phenotype. Discriminatory doses are highly effective in the case of a disruptive resistance mechanism such as that associated with SNPs at the target gene of the fungicide. The characterization of EC_50_ is required for fungicides associated with quantitative or semi-quantitative resistance mechanisms.

#### 7.2.2. Zoospore Microtiter Plate Assay

Microtiter testing procedures are based on the direct incubation of a sporangia suspension added to increasing concentrations of a fungicide. Such procedures are useful to investigate the inhibitory capacity of a fungicide on zoospores’ release and mobility, as in the case of the QoI compound famoxadone [84]. Fresh sporangia (final density 2.5 × 10^5^ sporangia/mL) harvested in cold water are added into a 96-well microtiter plate containing an aqueous suspension of fungicide at increasing concentrations. Quantification of sporangia germination is visually estimated by observing under microscope the release of zoospores 24 h after incubation at 20 °C in the dark (Figure 5B) and comparing the percentages calculated against those of the negative control [84,85,86]. However, the reliability of this method is limited since it does not consider a possible osmotic influence on sporangia germination caused by the direct addition of fungicides to the sporangia suspension.

#### 7.2.3. Oospore Assay

Bioassays on *P. viticola* oospores, the sexual and only overwintering structures of the pathogen, can be used to monitor resistance to all those fungicides affecting the differentiation or germination of these structures. The frequency of mutations conferring resistance to some fungicides can fluctuate during the growing seasons, as in the case of carboxylic acid amides [4]. This test can give an overview of the fungicide resistance state before the occurrence of primary infections, thus allowing a better understanding of the dynamics in the pathogen population and of the extent of selection pressure applied during the previous growing season. The test on oospores implies the collection of samples by randomly sampling leaves showing mosaic symptoms at the end of the grapevine growth season. Leaf fragments rich in oospores are cut out from leaves and placed inside nylon bags (pore size 100 μm) subjected to overwintering in vineyards or in controlled conditions. Germination assays are generally carried out three–five months after the start of overwintering. Fragments are ground in a glass mortar, then filtered through two nylon filters to separate oospores from leaf material (100 and 45 μm), and finally, resuspended in water. The suspension is inoculated and incubated in the dark at 20 °C on water agar plates (1%) containing increasing concentrations of fungicide (Figure 5C). By scoring the frequency of germinated oospores compared to the untreated control, it is possible to quantify the percentage of resistant individuals at a discriminatory fungicide concentration (quantitative evaluation of resistance) [4,66,67,87].

## 8. Molecular Assays

For fungicide classes with established molecular mechanisms of resistance, several molecular techniques can be applied for SNP(s) detection in the target gene. Most of the molecular technologies refer to PCR (polymerase chain reaction) and have the advantage of being more rapid and less expensive than biological assays. Besides pure detection, a resistant allele can be quantified with quite a low detection limit in a pathogen population [64]. The major issue related to molecular monitoring is the need to have a clear understanding of the resistance mechanisms, which is available for only a few fungicide classes. As a consequence, only the well-known resistance alleles can be monitored [62]. Consequently, molecular assays cannot be used to establish a baseline, and the concepts such as baseline and sensitivity shifting are replaced by the frequency distribution of resistant mutants within a fungal population [55].

The frequency of resistant individuals is extremely low during the initial phases of resistance evolution; therefore, molecular testing represents a useful tool to detect fungicide resistance when conventional bioassays are not able to do so [64,88]. Many advanced molecular tools such as denaturated high performance liquid chromatography (DHPLC), PCR, PCR-restriction fragment length polymorphism (PCR-RFLP), allele specific PCR, allele specific real-time PCR, and droplet digital PCR have been employed with success in the molecular detection of fungicide resistance for different plant pathogens for many years [29,89]. However, the mode of action of the fungicide, the relative resistance mechanism, and the SNPs associated with resistance [62] have to be known to run these testing procedures. In the specific case of *P. viticola*, these tests are at present available only for monitoring resistance to quinone outside inhibitors (QoIs) [90,91,92], carboxylic acid amides (CAAs) [4,93,94], and more recently, for quinone inside inhibitors (QiIs) and for quinone inside-outside inhibitors (QioIs) [73,95,96]. For other fungicide classes, the mechanisms of resistance are unknown or can potentially involve several genes, greatly complicating the development of molecular tools.

For QoIs and CAAs, resistance mechanisms in *P. viticola* are thoroughly documented [97,98,99]. This has made possible the development of a range of molecular methods. The resistance mechanism to QoI is due to SNPs in the cytochrome b gene [90,91,100]. The mutations associated with a shift in sensitivity reported so far are F129L, G137R, and G143A [97]. Currently, in *P. viticola* isolates, the resistance traits are associated only with F129L or G143A [99,101]. The percentage of individuals carrying F129L is significantly lower than the percentage of G143A, which is more widespread and is associated with a particularly high resistance factor [67,90]. As regards CAAs, a decrease of sensitivity to the fungicide is associated with several SNPs in the third gene of the cellulose synthase complex (*CesA3*). The resistance locus is present in codon 1105 of the *PvCesA3* gene of *P. viticola* and is characterized by a substitution of a glycine (G1105, codon CGC) with a different amino acid [98]. In European *P. viticola* populations, two possible allelic variants have been detected: the first involves the substitution of glycine with serine (G1105S, codon AGC) and the second one of glycine with valine (G1105V, codon GTG) at position 1105 in the deduced amino acid sequence [98,102]. G1105V is more rarely reported, and most of the time, it is the G1105S mutation that confers resistance to CAAs [4].

Rapid molecular testing procedures, aiming at detecting resistance to QoIs and CAAs, have been developed by using PCR-restriction fragment length polymorphism (RFLP) assays [39,103] and real-time PCR assays [68,104]. Compared to the time-consuming bioassays cited above, these PCR based assays can process a large number of *P. viticola* samples simultaneously and quickly became a common tool for the detection and evaluation of fungicide resistance for these two fungicide classes in *P. viticola* isolates. It must be pointed out that PCR-RFLP testing procedures have some intrinsic disadvantages, as an additional restriction enzyme digestion step after PCR amplification is required. To optimize the analytical time and to improve accuracy, the amplification-refractory mutation system PCR assay (ARMS) was developed to detect simultaneously the presence of CAAs and QoI resistant alleles in *P. viticola* populations [105]. With this method, the time for detection of mutations is reduced, because no restriction enzyme digestion is required. Unfortunately, this simple and rapid method for the simultaneous detection of *P. viticola* isolates resistant to QoIs and CAAs has some limitations because it can only detect the presence of the resistant alleles and is not able to distinguish between homozygous and heterozygous strains [42]. Due to the diploid nature of *P. viticola*, mutations in the coding sequence of genes do not necessarily cause mutant phenotypes. In the case of *PvCesA3*, the resistant G1105S/V character mentioned above is recessive, and it occurs twice in homozygous individuals (−/−) or once in heterozygous ones (−/+) [94,98]. For this reason, the use of two parallel PCR assays is required to discriminate between sensitive (−/+) and resistant (−/−) CAA isolates, doubling the workload. To overcome this issue and detect the presence of CAA-resistant strains of *P. viticola* in a single PCR reaction step, a tetra-primer PCR assay (ARMS) was applied to discriminate between homozygous and heterozygous strains (Zhang et al., 2017). In this PCR method, two pairs of primers are present in a single reaction that generates amplicons of different sizes, which allow one to distinguish the presence of two alleles in a single vial: one primer pair is specific for the mutation, and the other one consists of outer primers necessary to create a control band. However, the employment of two sets of primers in one reaction might in some cases lead to cross-amplification and false positives [106,107]. To solve this problem and to enhance specificity, sensitivity, and throughput in the detection of resistant and sensitive genotypes, a TaqMan-minor groove binding (MGB)-real time PCR was developed as a more decisive and precise tool [107].

## 9. Conclusions

The use of single-site fungicides for downy mildew control is closely related to the risk of the emergence of resistance. So far, *P. viticola* shows resistance to almost all fungicide classes. Monitoring represents the cornerstone of good resistance management, and the density and magnitude of collected data provide fundamental information about the risk of resistance emergence and spreading. Samples collected on a large scale, in commercial vineyards of different regions or in field trials where the application of the considered fungicide is repeated, could contribute to giving a global and unified vision of the resistance status. The sharing of monitoring results and the communication between public and industrial sectors have key roles in data interpretation and the formulation of recommendations for a sustainable and rational use of the products. The adaptation of *P. viticola* populations to the various selection pressures exerted in the vineyard by the different fungicide classes can be better understood with constant resistance monitoring through several years after resistance emergence in the field.

There is a great diversity among the testing procedures available for monitoring, and different information about the emergence and extension of resistance can be obtained using different methodologies. In the absence of molecular tools, biological tests remain fundamental in monitoring, and the degree of variation compared to a baseline sensitivity represents a good marker of changes in resistance. Considering the various resistance evolutionary phases and the complex life cycle of *P. viticola*, the information on the resistance phenomenon obtained with a single testing method is not sufficient. The mode of action of the fungicide under investigation, the characteristics of the targeted genetics, and the percentage of resistant strains in the investigated population can strongly influence the results, and the use of multiple testing procedures can help by providing a global and realistic view of resistance evolution.

## Figures and Tables

**Table 1:** List of antiperonosporic single/oligo-site active ingredients divided by chemical group, mechanism of action, and resistance reference. “CAA”, Carboxylic Acid Amide; “QoI”, Quinone outside Inhibitor; “QiI”, Quinone inside Inhibitor; “QioI”, Quinone inside-outside Inhibitor; “OSBPI”, Oxysterol-Binding Protein; ”-“not reported.

Group Name	Common Name	Chemical Group	Mode of Action	First Confirmed Resistance Reference	
				Report	Remarks
Cyanoacetamide-oxime	Cymoxanil	Cyanoacetamide-oxime	Unknown	Gullino et al., 1997	Reduced field performance
Phenylamides	Metalaxyl, Metalaxyl-M, Benalaxyl, Benalaxyl-M	Acylalanines	Inhibition of ribosomal RNA synthesis	Staub and Sozzi 1981; Bosshard and Schuepp 1983; Leroux and Clerjeau 1985	Reduced field performance
CAA	Dimethomorph	Cinnamic acid amides	Inhibition of cell wall biosynthesis	Gisi et al., 2007	Inheritance of resistance
	Iprovalicarb	Carbamate Vanilamides			
	Bentiavalicarb			Blum et al., 2010	Resistance mechanism
	Valifenalate				
	Mandipropamid	Mandelic acid amides			
QoI	Pyraclostrobin	Strobilurins	Inhibition of mitochondrial respiration, Complex III (Site Qo)	Heaney et al., 2000; Gullino et al., 2004	Reduced field performance
	Famoxadone	Oxazolidinone			
	Fenamidone	Imidazolones		Sierotzki et al., 2005	Review
QiI	Cyazofamid	Cyanoimidazole	Inhibition of mitochondrial respiration, Complex III (Site Qi)	Cherrad et al., 2018; Fontaine et al., 2019	Resistance mechanism
	Amisulbrom	Sulfonamide			
QioI	Ametoctradin	Triazolopyrimidine	Inhibition of mitochondrial respiration, Complex III (Sites Qi and Qo)	Mounkoro et al., 2018, Fontaine et al., 2019	Resistance mechanism
Benzamides	Zoxamide	Toluamides	Inhibition of cellular division	-	-
	Fluopicolide	Pyridinylmethylbenzamides	Delocalizes spectrin-like proteins	Note commune vigne 2020	Unknown mechanism
OSBPI	Oxathiapiprolin	Piperidinyl thianzole isoxazoline	Inhibition of oxysterol binding protein	-	-

## Data Availability

Data sharing not applicable.
